# Suicidal Thoughts and Self‐Harm Behavior During the COVID‐19 Pandemic in the United Kingdom: A Repeated Cross‐Sectional Population‐Based Survey Study

**DOI:** 10.1002/hsr2.72045

**Published:** 2026-04-12

**Authors:** Chiara Lombardo, Lijia Guo, Steven Martin, David Crepaz‐Keay, Martins Boss, Lucy Thorpe, Susan Solomon, Alec Morton, Gavin Davidson, Antonis A. Kousoulis, Tine Van Bortel

**Affiliations:** ^1^ Cambridge Public Health Interdisciplinary Research Centre, Department of Psychiatry University of Cambridge School of Clinical Medicine Cambridge UK; ^2^ Mental Health Foundation London UK; ^3^ Leicester School of Allied Health Sciences, Faculty of Health and Life Sciences De Montfort University Leicester UK; ^4^ Department of Management Science, Strathclyde Business School University of Strathclyde Glasgow UK; ^5^ School of Social Sciences, Education and Social Work Queen's University Belfast Belfast UK

**Keywords:** COVID‐19, lockdown, mental health, pandemic, risk factors, self‐harm, suicidal thoughts, United Kingdom

## Abstract

**Background and Aims:**

There are concerns that COVID‐19 and associated restrictive measures may have contributed to increased suicidal thoughts and self‐harm, despite contrasting scientific evidence. The objective of this study is to investigate how COVID‐19‐related restrictions affected suicidal thoughts and self‐harm in UK adults throughout the pandemic, to clarify the above issue and aid the design of targeted public mental health measures.

**Methods:**

Data from a representative, repeated cross‐sectional surveys with UK adults were evaluated between March 2020 and November 2021 (*n* = 48,996). Multivariate logistic regression models were used to quantify the association of lockdown periods with suicidal thoughts, self‐harm, and sociodemographic variables.

**Results:**

COVID‐19 and associated restrictive measures were associated with significantly increased prevalence and likelihood of reporting suicidal thoughts and self‐harm in young adults, people reporting a pre‐existing mental health condition, and people with disabilities. A general upward trajectory emerged over time in connection to suicidal thoughts and reporting self‐harm amongst specific groups, even during lockdowns lifting.

**Conclusion:**

Evidence from the study should guide a holistic public health response to future pandemics. Even when not linked to an increase in suicides, protecting the well‐being of people living in suicidal distress through programs that promote kindness, hope, and human dignity should be critical. Such actions can be taken in ways that do not compete with measures that prevent pandemics from spreading.

## 1 Introduction

There have been widespread concerns that the COVID‐19 pandemic, and measures taken to curb its spread, have had a negative impact on population mental health and suicide rates [1, 2]. Several mental health impacts have been observed, including: increased population‐level mental distress, mental ill‐health conditions and drug use; increased risk of developing a mental health condition following COVID‐19 infection; amplification of risk factors for poor mental health and well‐being; particular groups disproportionately experiencing mental health impacts associated with COVID‐19; and significant disruption to Public Mental Health services [3, 4, 5, 6]. However, most studies from various countries found no such elevated rates when suicidality pre‐COVID and post‐COVID were compared, apart from some specific groups [1, 7, 8, 9, 10]. Findings from systematic reviews suggest that, along with evidence on mental health disorders and suicide, COVID‐19 effects on mental health are nuanced and vary between groups and communities [11].

According to the DSM‐5, suicidal ideation is the act of thinking about, considering, or planning self‐harm or suicide, while self‐harm is defined as “self‐injury or self‐poisoning irrespective of the apparent purpose of the act.” In the DSM‐5 and earlier versions of the manual, suicide and suicidal behaviors (including self‐harm and suicidal ideation) are conceptualized primarily as a specific symptom of Major Depressive Disorder (MDD) and Borderline Personality Disorder (BPD), or as a possible negative consequence of other psychiatric diagnoses. Although suicides are usually associated with psychiatric illness, considerable evidence suggests that a significant number of individuals who die by suicide do not have diagnosable psychiatric disorders. In addition, the majority of people who complete suicide are not known to mental health services. Hubers et al. [12] show that suicidal ideation is a risk factor for completed suicide.

Before the COVID‐19 pandemic, suicidal thoughts were already a significant issue in the UK population. Previous research established that in 2014, the prevalence of lifetime suicidal thoughts in the United Kingdom was around 20.6%, and 5.4% of people reported having had suicidal thoughts during the past year [13, 14]. In March 2020, the UK government announced the first nationwide lockdown to stop the coronavirus spread. Several public health measures, such as stringent social restrictions, were imposed [4]. Initial research described significant increases in depression, anxiety, and suicide‐related behavior. A meta‐analytic estimate of suicidal ideation based on a large sample from different countries and populations identified that suicidal ideation rates during the COVID‐19 pandemic were higher than those reported in studies on general populations pre‐pandemic [15]. However, by the end of 2020, there was no such mental health “epidemic,” suicide rates remained unchanged or declined, and the extent of adverse psychological and social effects of the pandemic varied across countries [16]. Pirkis et al. [17] found that in high‐ and upper‐middle‐income countries, suicide numbers remained largely unchanged or declined in the early months of the pandemic. After 15 months, the suicide rates in most of the 33 data‐producing countries remained static and in some cases declined [9]. According to the Office of National Statistics (ONS), suicide occurrence rates in 2021 were lower than rates from 2017 to 2020 [18]. This coincides with the strictest lockdown periods of 2021 in England and Wales. Deisenhammer and Kemmler [19] found a significant decrease in suicide numbers during the first 6 months of the COVID‐19 pandemic compared to respective periods of the preceding year in Austria. However, a longitudinal study by Gimenez‐Palomo et al. [20] in Spain revealed a steady increase in self‐harm and suicidal behavior over a 3‐year period during the pandemic, highlighting the enduring psychological toll of prolonged social isolation, economic uncertainty, and health‐related anxiety.

Recent studies have highlighted critical shifts in both the healthcare landscape and mental health trends over the past few years. Honorato‐Cia et al. [21] conducted a bibliometric analysis examining Long COVID research between 2020 and 2024, showing a growing volume of studies focused on the long‐term effects of SARS‐CoV‐2 infections and their associated impacts on healthcare systems. Their findings underscore the evolution of research on chronic symptoms and the changing focus of therapeutic and public health strategies [21] and reflect the global urgency to understand and address the long‐term consequences of the virus. This increase in research supports the broader shift in public health priorities and the need for sustained surveillance of both physical and mental health outcomes.

In parallel, the Pan American Health Organization [22] has reported a marked increase in epidemic alerts related to respiratory viruses, including influenza, RSV, and emerging coronaviruses. These alerts reflect not only heightened viral activity but also improved detection and reporting mechanisms post‐COVID. The growing frequency of such alerts has raised concerns about the psychosocial stressors associated with recurring health threats and their potential to exacerbate mental health vulnerabilities.

Looking ahead, the VACCELERATE initiative, as detailed by Salmanton‐García et al. [23], has proposed a strategic framework for anticipating and mitigating future pandemics. Their ranking of the WHO's Blueprint priorities emphasizes the importance of early detection, rapid response, and mental health integration in epidemic preparedness. This aligns with the urgent need to contextualize suicide trends within a broader landscape of evolving public health strategies.

Although suicidal behaviors during the COVID‐19 pandemic have been extensively analyzed and discussed, only a few studies have evaluated the hypothesis that worsening risks of self‐harm and suicidal ideation are attributable to lockdowns [24]. The authors found an increase in self‐harm cases during and after the lockdown period. Furthermore, most studies on the impact of COVID‐19 lockdowns on suicidal ideation and self‐harm focused on the initial part of the pandemic or were conducted outside the United Kingdom [24, 25]. A longitudinal analysis of suicidal trends during the COVID‐19 in Spain showed how an annual mortality from the pandemic years was significantly higher than mortality from the pre‐pandemic ones (*p* < 0.01). Other non‐UK studies found that suicidal ideation was prevalent and increased significantly over time during lockdowns, with considerable variability between countries [26, 27, 28]. Our own repeated cross‐sectional survey data from the mixed‐methods “Coronavirus: Mental Health in the Pandemic” study found that key indicators of distress among UK adults worsened during the first 9 months of the pandemic, with a specific impact on some socio‐demographic groups.

In the present study, the main objective is to investigate how various COVID‐19‐related restrictions affected suicidal thoughts and self‐harm in UK adults throughout the pandemic, with the aim of aiding the design of appropriate targeted public mental health interventions and preventative implementation measures.

This study significantly contributes to the Sustainable Development Goals (SDGs) [29] by offering critical evidence that can guide policy development, intervention design, and the implementation of targeted mental health services. By examining the impacts of COVID‐19 restrictions on suicidal thoughts and self‐harm in UK adults, the study highlights the importance of evidence‐based strategies that can address mental health crises during and after large‐scale public health emergencies. The findings offer essential insights for governments and public health organizations to tailor mental health policies that are not only responsive to immediate needs but also preventative in nature.

## 2 Methods

### 2.1 Study Design and Data

**Figure 1:**
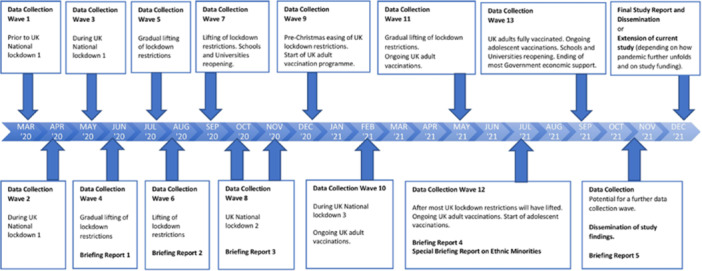
Data collection across all 13 waves.

This quantitative and survey‐based study is a sub‐study that forms part of a larger UK‐wide mixed‐methods consortium study entitled *Coronavirus: Mental Health in the Pandemic*, which aimed to investigate the mental health impacts of the Coronavirus pandemic in UK adults, taking a public health perspective. The researchers used an online repeated cross‐sectional population survey, with non‐probability quota sampling, repeated over 13 waves from March 2020 to November 2021. The inclusion criteria of the sample are the adult population (18+ years with no upper age limit) from across the entire UK (England, Wales, Scotland, and Northern Ireland) and from all walks of life. Each wave selected a sample of 4000 adults (> 18) living in the United Kingdom, designed to be representative of the population based on age, sex, education, and social class compared with national statistics (refer to the detailed methodology described elsewhere in Van Bortel et al. [30]). In total, there were 51,122 participants across all 13 waves (Figure 1). However, we omitted Wave 1 participants as they were not asked questions about suicidal thoughts/self‐harm.

Thus, in this study, repeated cross‐sectional data were used from surveys undertaken between April 2, 2020, and November 19, 2021 (Box 1). Participants who did not respond to suicidal thoughts or self‐harm questions were also excluded. The final sample consisted of 48,996 participants across 12 waves, over a period of 18 months. Thus, the final sample included in this study was quota‐balanced and weighted to distributions of the UK adult population (> 18) at each time point of data collection across the 12 waves from April 2020 to November 2021.

Box 1: **Cross‐sectional surveys undertaken with adults in the United Kingdom.**

**Table:** 

Waves	Dates
*Wave 1*	N.A. (no relevant data on suicidal thoughts/self‐harm collected)
	From March 17 to 18, 2020
	Prior to Lockdown 1
*Wave 2*	From April 2 to 3, 2020 During Lockdown 1
*Wave 3*	From April 24 to 26, 2020 During Lockdown 1
*Wave 4*	From May 28 to 29, 2020 During lifting of Lockdown 1
*Wave 5*	From June 18 to 22, 2020 During lifting of Lockdown 1
*Wave 6*	From July 30 to August 3, 2020 During lifting of Lockdown 1
*Wave 7*	From August 26 to 28, 2020 During lifting of Lockdown 1
*Wave 8*	From November 26 to 30, 2020 During Lockdown 2
*Wave 9*	From December 21 to 23, 2020 During lifting of Lockdown 2
*Wave 10*	From February 24 to 26, 2021 During Lockdown 3
*Wave 11*	From June 18 to July 2, 2021 During lifting of Lockdown 3
*Wave 12*	From September 9 to 16, 2021 Lockdowns lifted
*Wave 13*	From November 10 to 19, 2021 Lockdowns lifted

*Note:* We did not collect these data in Wave 1, so this study used data from Waves 2 to 13, containing information on suicidal thoughts and self‐harm.

### 2.2 Measuring Self‐Reported Suicidal Thoughts and Self‐Harm

#### 2.2.1 Confidentiality Statement

Participants signed up for the survey via YouGov after agreement of terms, conditions, and privacy policy [31]. Before responding to the online mental health survey, individuals were asked if they would like to participate in the section, before suicidal thoughts were assessed (Box 2).

Box 2: **Questions on suicidal thoughts.**

**Table:** 

Question	Answers
*Are you happy to participate in this section of a survey*	“Yes” or “No”
*Have you experienced suicidal thoughts/feelings in the past 2 weeks as a result of the Coronavirus (COVID‐19) pandemic?*	“Yes”, “No”, or “Prefer Not to Say”
*Have you deliberately hurt yourself in the past 2 weeks as a result of the Coronavirus (COVID‐19) pandemic?*	“Yes”, “No”, or “Prefer Not to Say”
*How often have you done each of the following as a result of the Coronavirus (COVID‐19) pandemic in the past 2 weeks?*	“Once a day or more often”, “Nearly every day”, “A few times a week”, “Passing thoughts”, “Don't know” or “Prefer not to say”

### 2.3 Independent Variables

The study included restriction variables as follows: Waves 2–3 (Lockdown 1), Waves 4–7 (Lifting of Lockdown 1), Wave 8 (Lockdown 2), Wave 9 (Lifting of Lockdown 2), Wave 10 (Lockdown 3), and Waves 11–13 (Lifting of Lockdown 3). Sociodemographic variables were also included (Box 3), as these variables could be important influencing factors of individuals’ mental health and well‐being.

Box 3: **Sociodemographic variables.**

**Table:** 

Question	Answers
*Sex*	“Male” or “Female”
*Age groups*	“18‐24”, “25‐34”, “35‐44”, “55+”
*Working status*	“unemployed”, “full‐time job”, “part‐time job”, “retired”, “not working” and “full‐time student”
Social grade*	ABC1 and C2DE
*Marital status*	“married”, “in a civil partnership”, “separated but still legally married or in a civil partnership”, “living with a partner but neither married nor in a civil partnership”, “in a relationship, but not living together”, “single”, “divorced”, “widowed”
*Pre‐existing mental health condition*	“yes” or “no”
*Long‐term condition*	“limited a lot”, “limited a little” or “no”

*We used the NRS Social grades ABC1 (corresponding to the upper‐middle to lower‐middle class) and C2DE (skilled working class to individuals at the lowest level of subsistence).

### 2.4 Statistical Analysis

First, the prevalence of suicidal thoughts/self‐harm across various groups was presented. Afterward, multivariate regression analysis using unadjusted and adjusted models was conducted. The researchers first performed logistic regression analyses with suicidal thoughts and self‐harm as dependent variables, followed by either Wave dummy or Restriction variables and other sociodemographic controls. Further, interaction terms were included (restrictions#socio‐demographic controls), based on the previous main models, to detect how various population groups might be affected. In these models, the aforementioned sociodemographic variables are still included in the model analysis, with the addition of the interaction terms of “restriction period” and the three variables “age, working status, and gender.” The interaction effects of all the sociodemographic variables were also tested and found that these three aspects have significant moderating effects and thus presented these aspects. Results for multivariate logistic regressions were reported in odds ratios (ORs). For each outcome/covariate, “Prefer not to say/Don't know” responses were excluded from analysis, and observations with missing covariates were dropped listwise for each model. Sample weights were used to ensure UK representativeness. The a priori level of significance was set at the level of 5%, and tests were conducted as two‐sided tests to consider either direction of the effect. All analyses were conducted using STATA (version 16.0).

### 2.5 Ethics Approvals

Ethics approvals were obtained from the University of Cambridge Psychology Research Ethics Committee (No. PRE 2020.050) and De Montfort University Faculty of Health and Life Sciences Research Ethics Committee (No. REF 422991).

## 3 Results

### 3.1 Descriptive Analysis: Trends in Suicidal Thoughts and Self‐Harm

#### 3.1.1 Suicidal Thoughts

**Figure 2:**
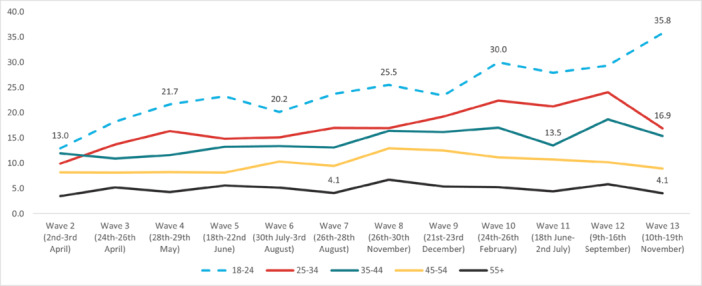
Prevalence of suicidal thoughts and age groups. *Note:* The graph above illustrates the prevalence of suicidal thoughts by age group in percentages, and the values are survey weighted.

**Figure 3:**
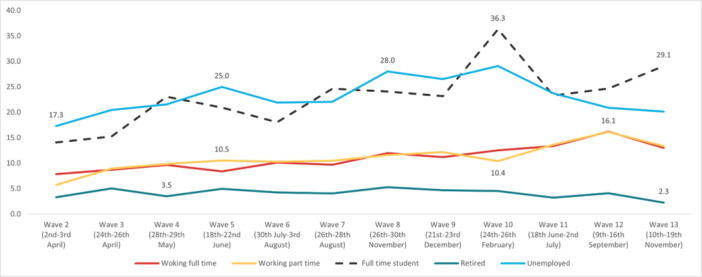
Prevalence of suicidal thoughts and working status. *Note:* The graph above illustrates the prevalence of suicidal thoughts by work status in percentages, and the values are survey weighted.

**Figure 4:**
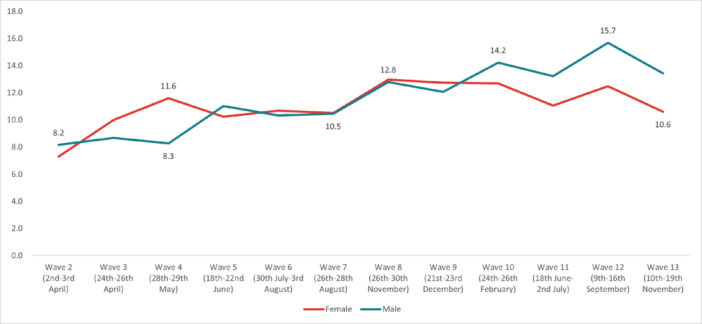
Prevalence of suicidal thoughts and gender. *Note:* The graph above illustrates the prevalence of suicidal thoughts by gender in percentages, and the values are survey weighted.

**Figure 5:**
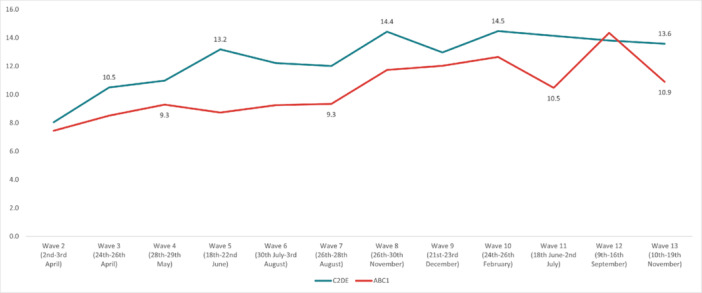
Prevalence of suicidal thoughts and social grade. *Note:* The graph above illustrates the prevalence of suicidal thoughts by social grade in percentages, and the values are survey weighted.

**Figure 6:**
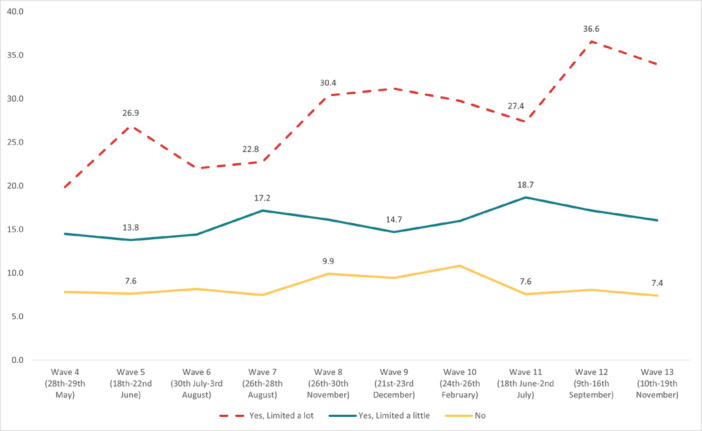
Prevalence of suicidal thoughts and disability. *Note:* The graph above illustrates the prevalence of suicidal thoughts by disability in percentages, and the values are survey weighted.

**Figure 7:**
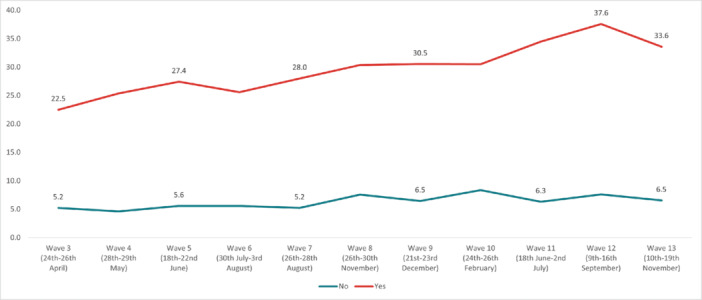
Prevalence of suicidal thoughts and existing mental health conditions. *Note:* The graph above illustrates the prevalence of suicidal thoughts by existing mental health conditions in percentages, and the values are survey weighted.

**Figure 8:**
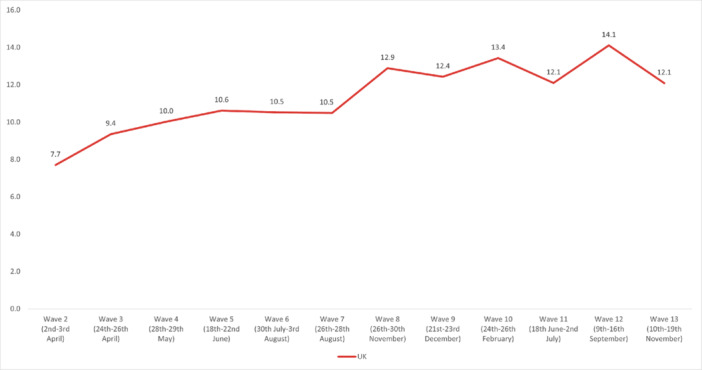
Prevalence of suicidal thoughts and the United Kingdom total. *Note:* The graph above illustrates the prevalence of suicidal thoughts and the United Kingdom in percentages, and the values are survey weighted.

The highest overall prevalence of *suicidal thoughts* in the United Kingdom was experienced in Wave 12 (14.1%) and the lowest in Wave 2 (7.7%). The highest prevalence by age group was reported by 18‐ to 24‐year‐olds in Wave 13 (35.8%), and the lowest in 55+ year‐olds in Wave 2 (3.49%). By work status, the highest prevalence was reported by full‐time students in Wave 10 (36.3%), and the lowest was in the retired group in Wave 13 (2.25%). The highest prevalence by gender was reported by males in Wave 12 (15.7%); while social grade C2DE (skilled or semi‐skilled manual workers, state pensioners, unemployed with state benefits only, casual workers) reported the highest prevalence in Wave 10 (14.5%). Moreover, in the marital status group, those in a civil partnership reported the highest prevalence of suicidal thoughts in Wave 12 (34%), while widowed people reported the lowest in Wave 13 (2.8%). Furthermore, individuals with pre‐existing mental health conditions and those with long‐term conditions reported a higher prevalence of experiencing suicidal thoughts, with the highest prevalences for both groups in Wave 12 (37.6% and 36.6%, respectively) (Figures 2, 3, 4, 5, 6, 7, 8).

#### 3.1.2 Self‐Harm

**Figure 9:**
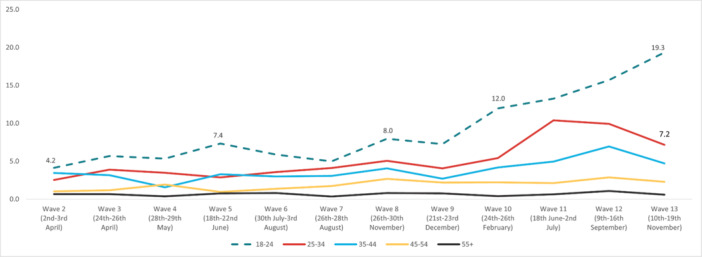
Prevalence of self‐harm and age groups total. *Note:* The graph above illustrates the prevalence of self‐harm by age groups in percentages, and the values are survey weighted.

**Figure 10:**
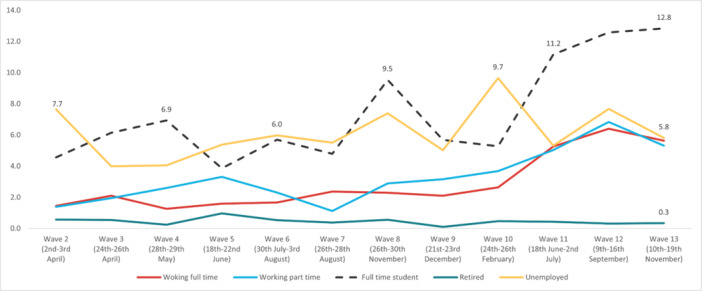
Prevalence of self‐harm and work status total. *Note:* The graph above illustrates the prevalence of self‐harm by work status in percentages, and the values are survey weighted.

**Figure 11:**
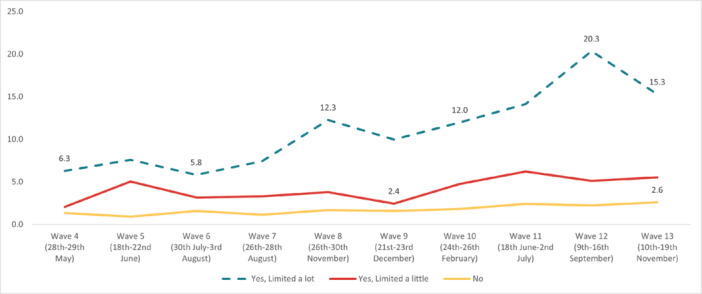
Prevalence of self‐harm and disability. *Note:* The graph above illustrates the prevalence of self‐harm by disability in percentages, and the values are survey weighted.

**Figure 12:**
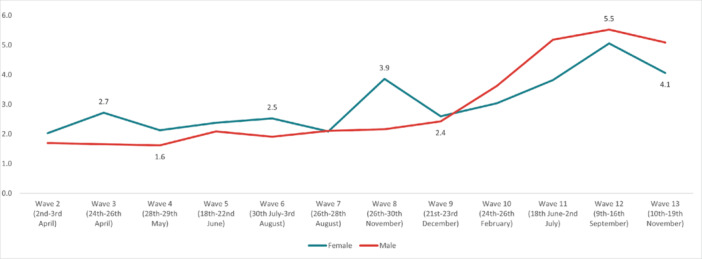
Prevalence of self‐harm and gender. *Note:* The graph above illustrates the prevalence of self‐harm by gender in percentages, and the values are survey weighted.

**Figure 13:**
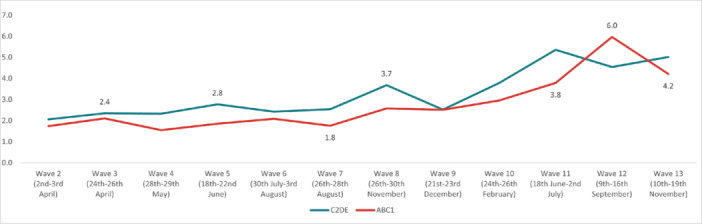
Prevalence of self‐harm and social grade. *Note:* The graph above illustrates the prevalence of self‐harm by social grade in percentages, and the values are survey weighted.

**Figure 14:**
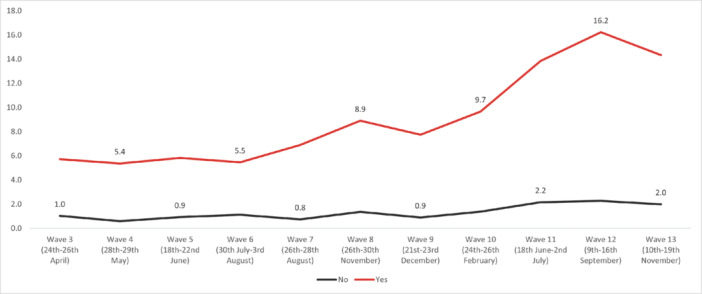
Prevalence of Self‐harm and existing Mental Health Conditions. *Note:* The graph above illustrates the prevalence of self‐harm and existing mental health conditions and the values are survey weighted.

**Figure 15:**
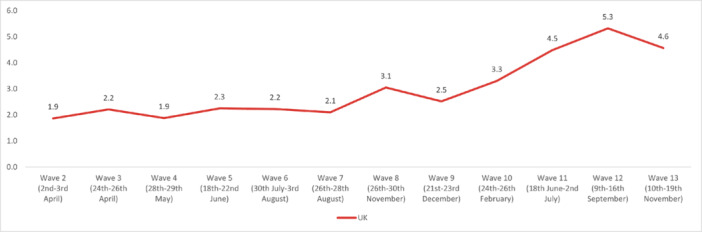
Prevalence of self‐harm and the United Kingdom total. *Note:* The graph above illustrates the prevalence of self‐harm and the United Kingdom in percentages, and the values are survey weighted.

In regard to *self‐harm*, the highest overall prevalence was experienced in Wave 12 (5.32%) and the lowest in Wave 2 (1.87%). The highest prevalence by age was reported by 18‐ to 24‐year‐olds in Wave 13 (19.3%), and the lowest in the 55+ age group in Wave 7 (0.35%). The highest prevalence by work status was reported by full‐time students in Wave 13 (12.8%), with the lowest in the retired group in Wave 9 (0.1%). By gender, the highest prevalence was reported by males in Wave 12 (5.52%), while the social grade ABC1 (managerial, administrative, and professional workers) reported the highest prevalence (5.97%) in Wave 12. Furthermore, individuals with pre‐existing mental health conditions reported experiencing suicidal thoughts more often, with the highest prevalence reported in Wave 12 (16.2%). Finally, people with long‐term conditions, who were physically significantly limited, reported a higher prevalence of suicidal thoughts compared to people without long‐term conditions, with the highest prevalence during Wave 12 (20.3%) (Figures 9, 10, 11, 12, 13, 14, 15).

### 3.2 Multivariate Analysis

Two multivariate logistic regression models for this analysis were used. In the first model, suicidal thoughts/self‐harm were used as dependent variables and, subsequently, wave/restriction, age, sex, working status, and social grade sociodemographic variables. There were a small number of missing values in the sex variable (*n* = 27). In the second model, the researchers added pre‐existing mental health conditions (*n* = 44,018), disabilities (*n* = 42,403), and marital status variables (*n* = 42,074). However, since questions about these variables were asked only from Waves 3 to 4 onwards, the researchers had to drop observations from Waves 2 and 3 in this model.

#### 3.2.1 Suicidal Thoughts

##### 3.2.1.1 Changing Trends of Suicidal Thoughts

Multivariate logistic regression was used to explore the associations between various time points, sociodemographic covariates, and suicidal thoughts (Table 1). In the first model, unadjusted analysis illustrated that the OR for all study waves was significantly higher than 1, with Wave 12 being the highest (OR = 2; 95% confidence interval [CI] = 1.6–2.4). In addition, the 18–24 age group (OR = 6.2; 95% CI = 5.6–6) and the unemployed (OR = 2.5; 95% CI = 2.2–2.7) were associated with the highest odds of experiencing suicidal thoughts. After adjusting for various control variables, similar effect sizes were noticed, with the exception of the full‐time student group OR being reduced (OR = 1.3; 95% CI = 1.1–1.5).

After examining the second model, the unadjusted analysis showed a similarly significant effect size. Analyses showed that the odds of reporting suicidal thoughts were significantly higher among individuals with pre‐existing mental health conditions, with an OR of 6.2 (95% CI = 5.8–6.6). In addition, having a long‐term disability was associated with higher odds of experiencing suicidal thoughts (OR = 4.3; 95% CI = 3.9–4.7). Finally, individuals who reported being widowed (OR = 0.9; 95% CI = 0.7–1.1) were not significantly associated with an increase in suicidal thoughts compared to other marital status groups in the unadjusted analysis. However, the adjusted analysis showed that individuals who were widowed had an OR of 1.4 (95% CI = 1.1–1.8) for reporting suicidal thoughts.

**Table 1:** The changing trends of suicidal thoughts over time (by wave).

Outcome	Variable	Category	Unadjusted model			Adjusted model		
			OR	*p*	95% CI	OR	*p*	95% CI
Suicidal thoughts	Wave							
		Wave 3	1.2	0.037	1–1.5	1.2	0.051	0.9–1.5
		Wave 4	1.3	0.005	1.1–1.6	1.3	0.009	1.1–1.6
		Wave 5	1.4	0.001	1.2–1.7	1.4	0.002	1.1–1.7
		Wave 6	1.4	0.001	1.2–1.7	1.4	0.001	1.1–1.7
		Wave 7	1.4	0.001	1.2–1.7	1.4	0.001	1.2–1.7
		Wave 8	1.8	0.001	1.5–2.2	1.8	0.001	1.5–2.2
		Wave 9	1.7	0.001	1.4–2.1	1.7	0.001	1.4–2.1
		Wave 10	1.9	0.001	1.5–2.3	1.9	0.001	1.5–2.3
		Wave 11	1.7	0.001	1.4–2	1.7	0.001	1.4–2
		Wave 12	2	0.001	1.6–2.4	2	0.001	1.6–2.4
		Wave 13	1.6	0.001	1.4–2	1.6	0.001	1.3–2
	Sex	Male	1	0.224	0.9–1.1	1.1	0.002	1–1.2
	Age (years)	18–24	6.2	0.001	5.6–6.8	4.9	0.001	4.3–5.6
		25–34	4	0.001	3.7–4.4	3.4	0.001	3.1–3.9
		35–44	3.2	0.011	2.9–3.5	2.6	0.001	2.3–2.9
		45–54	2.1	0.001	1.9–2.3	1.7	0.001	1.5–1.9
	Working status	Working part‐time	1.3	0.536	0.9–1.1	1.2	0.001	1.1–1.3
		Full‐time student	2.4	0.001	2.1–2.7	1.3	0.002	1.1–1.5
		Retired	0.3	0.001	0.3–0.4	0.8	0.007	0.7–1
		Unemployed	2.5	0.001	2.2–2.7	2.1	0.001	1.9–2.4
	Social grade	ABC 1, middle class	0.8	0.001	0.8–0.9	0.8	0.001	0.8–0.9
Suicidal thoughts	Wave	Wave 4 (reference)						
		Wave 5	1.4	0.001	1.2–1.7	1.1	0.133	1–1.4
		Wave 6	1.4	0.001	1.2–1.7	1.1	0.177	0.9–1.3
		Wave 7	1.4	0.001	1.2–1.7	1.1	0.146	1–1.4
		Wave 8	1.8	0.001	1.5–2.2	1.5	0.001	1.3–1.8
		Wave 9	1.7	0.001	1.4–2.1	1.3	0.001	1.1–1.6
		Wave 10	1.9	0.001	1.5–2.3	1.6	0.001	1.3–1.9
		Wave 11	1.7	0.001	1.4–2	1.1	0.252	0.9–1.4
		Wave 12	2	0.001	1.6–2.4	1.5	0.001	1.2–1.7
		Wave 13	1.6	0.001	1.4–2	1.3	0.004	1.1–1.5
	Sex	Male	1	0.224	0.9–1.1	1.2	0.001	1.1–1.3
	Age (years)	18–24	6.2	0.001	5.6–6.8	4.3	0.001	3.6–5.1
		25–34	4	0.001	3.7–4.4	3.3	0.001	2.9–3.8
		35–44	3.2	0.011	2.9–3.5	2.5	0.001	2.2–2.9
		45–54	2.1	0.001	1.9–2.3	1.5	0.001	1.3–1.8
	Working status	Working part‐time	1.3	0.536	0.9–1.1	1	0.954	0.9–1.1
		Full‐time student	2.4	0.001	2.1–2.7	1.1	0.203	0.9–1.4
		Retired	0.3	0.001	0.3–0.4	0.7	0.001	0.6–0.8
		Unemployed	2.5	0.001	2.2–2.7	1.2	0.004	1.1–1.4
	Social grade	ABC 1, middle class	0.8	0.001	0.8–0.9	1	0.001	1.1–1.3
	Marital status	In a civil partnership	3.2	0.001	2.5–4.1	2	0.001	1.4–2.6
		Separated but still legally married or in a civil partnership	3	0.001	2.6–3.5	1.8	0.001	1.4–2.1
		Living with a partner but neither married nor in a civil partnership	1.8	0.001	1.6–2	1	0.546	0.9–1.2
		In a relationship but not living together	3.1	0.001	2.7–3.5	1.4	0.001	1.2–1.7
		Single	3.1	0.001	2.9–3.4	1.5	0.001	1.3–1.6
		Divorced	1.4	0.001	1.2–1.5	1.5	0.001	1.3–1.7
		Widowed	0.9	0.243	0.7–1.1	1.4	0.004	1.1–1.8
	Pre‐existing mental health condition	Yes	6.2	0.001	5.8–6.6	3.8	0.001	3.5–4.2
	Disability/long‐term condition	Yes, limited a lot	4.3	0.001	3.9–4.7	3.2	0.001	2.9–3.6
		Yes, limited a little	2.1	0.001	1.9–2.2	1.9	0.001	1.7–2

##### 3.2.1.2 Association of Restrictions With Suicidal Thoughts

In the first model, the unadjusted analyses showed that in the UK population, the odds of reporting suicidal thoughts were higher among individuals in the second (OR = 1.5; 95% CI = 1.3–1.8) and third (OR = 1.6; 95% CI = 1.4–1.8) lockdowns compared to Lockdown 1 (reference period). More importantly, the lifting of these was associated with higher odds of experiencing suicidal thoughts: lifting Lockdown 1 (OR = 1.2; 95% CI = 1.1–1.3), lifting Lockdown 2 (OR = 1.5; 95% CI = 1.3–1.8), lifting Lockdown 3 (OR = 1.5; 95% CI = 1.4–1.7). There were no major differences in the adjusted model. Analysis in the second model for both unadjusted and adjusted models demonstrated similar results, with the second and third lockdowns being associated with increased suicidal thoughts. After analyzing restriction periods and related prevalence of suicidal thoughts, it was found that, compared to Lockdown 1, the second and third lockdown were associated with a higher likelihood for people having suicidal thoughts; whereas, lifting of lockdowns was also associated with higher likelihood (Table 2).

**Table 2:** Association of restrictions with suicidal thoughts.

Outcome	Variable	Category	Unadjusted model				Adjusted model		
			OR	*p*	95% CI		OR	*p*	95% CI
Suicidal thoughts	Wave	Lockdown 1 (reference)				Lockdown 1 (reference)			
		Lifting of Lockdown 1	1.2	0.001	1.1–1.3	Lifting of Lockdown 1	1.2	0.001	1.1–1.3
		Lockdown 2	1.5	0.001	1.3–1.8	Lockdown 2	1.6	0.001	1.4–1.8
		Lifting of Lockdown 2	1.5	0.001	1.3–1.7	Lifting of Lockdown 2	1.5	0.001	1.3–1.7
		Lockdown 3	1.6	0.001	1.4–1.8	Lockdown 3	1.6	0.001	1.4–1.9
		Lifting of Lockdown 3	1.5	0.001	1.4–1.7	Lifting of Lockdown 3	1.5	0.001	1.4–1.7
	Sex	Male	1	0.224	0.9–1.1	Male	1.1	0.002	1–1.2
	Age (years)	18–24	6.2	0.001	5.6–6.8	18–24	4.9	0.001	4.3–5.6
		25–34	4	0.001	3.7–4.4	25–34	3.4	0.001	3–3.8
		35–44	3.2	0.011	2.9–3.5	35–44	2.6	0.001	2.3–2.9
		45–54	2.1	0.001	1.9–2.3	45–54	1.7	0.001	1.5–1.9
	Working status	Working part‐time	1.3	0.536	0.9–1.1	Working part‐time	1.2	0.001	1.1–1.3
		Full‐time student	2.4	0.001	2.1–2.7	Full‐time student	1.3	0.002	1.1–1.5
		Retired	0.3	0.001	0.3–0.4	Retired	0.8	0.007	0.7–0.9
		Unemployed	2.5	0.001	2.2–2.7	Unemployed	2.2	0.001	1.9–2.4
	Social grade	ABC 1, middle class	0.8	0.001	0.8–0.9	ABC 1, middle class	0.9	0.001	0.8–0.9
Suicidal thoughts	Wave								
		Lifting of Lockdown 1				Lifting of Lockdown 1			
		Lockdown 2	1.5	0.001	1.3–1.8	Lockdown 2	1.4	0.001	1.2–1.5
		Lifting of Lockdown 2	1.5	0.001	1.3–1.7	Lifting of Lockdown 2	1.2	0.003	1.1–1.4
		Lockdown 3	1.6	0.001	1.4–1.8	Lockdown 3	1.4	0.001	1.3–1.7
		Lifting of Lockdown 3	1.5	0.001	1.4–1.7	Lifting of Lockdown 3	1.2	0.001	1.1–1.3
	Sex	Male	1	0.224	0.9–1.1	Male	1.2	0.001	1.1–1.3
	Age (years)	18–24	6.2	0.001	5.6–6.8	18–24	4.2	0.001	3.6–5
		25–34	4	0.001	3.7–4.4	25–34	3.3	0.001	2.9–3.8
		35–44	3.2	0.011	2.9–3.5	35–44	2.5	0.001	2.2–2.9
		45–54	2.1	0.001	1.9–2.3	45–54	1.5	0.001	1.3–1.7
	Working status	Working part‐time	1.3	0.536	0.9–1.1	Working part‐time	1.0	0.945	0.9–1.1
		Full‐time student	2.4	0.001	2.1–2.7	Full‐time student	1.1	0.205	0.9–1.4
		Retired	0.3	0.001	0.3–0.4	Retired	0.7	0.001	0.6–0.8
		Unemployed	2.5	0.001	2.2–2.7	Unemployed	1.2	0.004	1.1–1.4
	Social grade	ABC 1, middle class	0.8	0.001	0.8–0.9	ABC 1, middle class	1.0	0.647	0.9–1.1
	Marital status	In a civil partnership	3.2	0.001	2.5–4.1	In a civil partnership	1.9	0.001	1.4–2.6
		Separated but still legally married or in a civil partnership	3	0.001	2.6–3.5	Separated but still legally married or in a civil partnership	1.6	0.001	1.3–1.9
		Living with a partner but neither married nor in a civil partnership	1.8	0.001	1.6–2	Living with a partner but neither married nor in a civil partnership	1.0	0.665	0.9–1.2
		In a relationship but not living together	3.1	0.001	2.7–3.5	In a relationship but not living together	1.4	0.001	1.2–1.7
		Single	3.1	0.001	2.9–3.4	Single	1.5	0.001	1.3–1.6
		Divorced	1.4	0.001	1.2–1.5	Divorced	1.4	0.001	1.2–1.6
		Widowed	0.9	0.243	0.7–1.1	Widowed	1.4	0.005	1.1–1.8
	Pre‐existing mental health condition	Yes	6.2	0.001	5.8–6.6	Yes	3.8	0.001	3.5–4.2
	Disability/long‐term condition	Yes, limited a lot	4.3	0.001	3.9–4.7	Yes, limited a lot	3.2	0.001	2.9–3.6
		Yes, limited a little	2.1	0.001	1.9–2.2	Yes, limited a little	1.9	0.001	1.7–2.1

#### 3.2.2 Self‐Harm

##### 3.2.2.1 Changing Trends of Self‐Harm

The researchers used multivariate logistic regression to explore the associations between various time points, sociodemographic covariates, and self‐harm (Table 3). Unadjusted analysis from the first model illustrated that for study Waves 3–9, the OR for all waves was higher than 1, with the exception of Wave 4 (OR = 1; 95% CI = 0.7–1.5). However, the p‐values were not statistically significant, with the exception of Wave 8 (OR = 1.7; 95% CI = 1.1–2.5). On the other hand, the OR for Waves 11–13 was higher than 2, with Wave 12 being the highest (OR = 2.9; 95% CI = 2–4.3). Additionally, all age groups were associated with higher odds of experiencing self‐harm when compared to the 55+ age group, with the 18–24 group being the highest (OR = 15; 95% CI = 12.2–18.5). Finally, in the working status group, full‐time students (OR = 2.5; 95% CI = 2.1–3.1) and unemployed people (OR = 2.2; 95% CI = 1.8–2.7) were associated with higher odds of experiencing self‐harm when compared to the full‐time job group. Individuals in the retired working‐status group reported the lowest odds of experiencing self‐harm (OR = 0.2; 95% CI = 0.1–0.2). Similar results were observed in the adjusted model. Furthermore, in the second model, the unadjusted analysis showed a significant association for Waves 8, 10–13, with the OR higher than 1, with Wave 13 being the highest (OR = 2.9; 95% CI = 2–4.3).

Additionally, all age groups were associated with higher odds of experiencing self‐harm when compared to the 55+ age group, with the 18–24 group being the highest (OR = 15; 95% CI = 12.2–18.5). Finally, in the working status group, full‐time students (OR = 2.5; 95% CI = 2.1–3.1) and unemployed people (OR = 2.2; 95% CI = 1.8–2.7) were associated with higher odds of experiencing self‐harm when compared to the full‐time job group. Individuals in the retired working‐status group reported the lowest odds of experiencing self‐harm (OR = 0.2; 95% CI = 0.1–0.2). Similar results were observed in the adjusted model. Furthermore, in the second model, the unadjusted analysis showed a significant association for Waves 8, 10–13, with the OR higher than 1, with Wave 13 being the highest (OR = 2.9; 95% CI = 2–4.3).

**Table 3:** The changing trends of self‐harm over time (by wave).

Outcome	Variable	Category	Unadjusted model			Adjusted Model		
			OR	*p*	95% CI	OR	*p*	95% CI
Self‐harm	Wave	Wave 3	1.2	0.418	0.8–1.8	1.2	0.45	0.8–1.8
		Wave 4	1	0.973	0.7–1.5	1	0.922	0.6–1.5
		Wave 5	1.2	0.373	0.8–1.8	1.2	0.497	0.8–1.8
		Wave 6	1.2	0.382	0.8–1.8	1.2	0.428	0.8–1.8
		Wave 7	1.2	0.595	0.7–1.7	1.2	0.601	0.7–1.7
		Wave 8	1.7	0.013	1.1–2.5	1.7	0.01	1.1–2.6
		Wave 9	1.4	0.151	0.9–2.1	1.4	0.13	0.9–2.1
		Wave 10	1.8	0.005	1.2–2.7	1.8	0.006	1.2–2.7
		Wave 11	2.5	0.001	1.7–3.6	2.5	0.001	1.7–3.7
		Wave 12	2.9	0.001	2–4.3	3	0.001	2–4.4
		Wave 13	2.5	0.001	1.7–3.7	2.6	0.001	1.7–3.8
	Sex	Male	0.9	0.52	0.9–1.1	1	0.547	0.9–1.2
	Age (years)	18–24	15	0.001	12.2–18.5	12	0.001	9–16
		25–34	8.4	0.001	6.8–10.3	6.5	0.001	5–8.6
		35–44	5.8	0.001	4.6–7.2	4.4	0.001	3.3–5.8
		45–54	2.9	0.001	2.3–3.7	2.1	0.001	1.6–2.9
	Working status	Working part‐time	1.2	0.029	1–1.4	1.3	0.001	1.1–1.6
		Full‐time student	2.5	0.001	2.1–3.1	1.1	0.539	0.9–1.4
		Retired	0.2	0.001	0.1–0.2	0.6	0.028	0.4–1
		Unemployed	2.2	0.001	1.8–2.7	1.7	0.001	1.4–2.2
	Social grade	ABC 1, middle class	0.8	0.001	0.7–0.9	0.9	0.058	0.8–1
Self‐harm	Wave	Wave 4 (reference)						
		Wave 5	1.2	0.373	0.8–1.8	1.2	0.291	0.8–1.8
		Wave 6	1.2	0.382	0.8–1.8	1.4	0.1	0.9–2
		Wave 7	1.2	0.595	0.7–1.7	1.3	0.167	0.9–1.9
		Wave 8	1.7	0.013	1.1–2.5	2	0.001	1.4–2.8
		Wave 9	1.4	0.151	0.9–2.1	1.5	0.038	1–2.1
		Wave 10	1.8	0.005	1.2–2.7	2	0.001	1.4–2.8
		Wave 11	2.5	0.001	1.7–3.6	2.4	0.001	1.6–3.5
		Wave 12	2.9	0.001	2–4.3	2.6	0.001	1.9–3.6
		Wave 13	2.5	0.001	1.7–3.7	2.6	0.001	1.8–3.5
	Sex	Male	0.9	0.52	0.9–1.1	1.1	0.071	1–1.3
	Age (years)	18–24	15	0.001	12.2–18.5	11.6	0.001	8.3–16.1
		25–34	8.4	0.001	6.8–10.3	6.2	0.001	4.6–8.4
		35–44	5.8	0.001	4.6–7.2	4.1	0.001	3–5.5
		45–54	2.9	0.001	2.3–3.7	1.8	0.001	1.3–2.5
	Working status	Working part‐time	1.2	0.029	1–1.4	1.1	0.395	0.9–1.3
		Full‐time student	2.5	0.001	2.1–3.1	1	0.877	0.7–1.3
		Retired	0.2	0.001	0.1–0.2	0.4	0.001	0.3–0.7
		Unemployed	2.2	0.001	1.8–2.7	0.9	0.556	0.7–1.2
	Social grade	ABC 1, middle class	0.8	0.001	0.7–0.9	1	0.6	0.9–1.2
	Marital status	In a civil partnership	4.5	0.001	3.1–6.4	1.6	0.031	1–2.4
		Separated but still legally married or in a civil partnership	3.4	0.001	2.6–4.5	1.1	0.584	0.8–1.6
		Living with a partner but neither married nor in a civil partnership	2	0.001	1.6–2.4	0.9	0.308	0.7–1.1
		In a relationship but not living together	3.4	0.001	2.7–4.3	1	0.801	0.8–1.4
		Single	2.8	0.001	2.4–3.3	0.9	0.303	0.7–1.1
		Divorced	1.4	0.003	1.1–1.8	1.3	0.102	1–1.7
		Widowed	0.4	0.004	0.2–0.8	1	0.906	0.5–1.8
	Pre‐existing mental health condition	Yes	7.3	0.001	6.4–8.3	3.6	0.001	3–4.3
	Disability/long‐term condition	Yes, limited a lot	7.5	0.001	6.5–8.7	6.1	0.001	5–7.5
		Yes, limited a little	2.6	0.001	2.2–3	2.2	0.001	1.8–2.7

##### 3.2.2.2 Association of Restrictions With Self‐Harm

In the first model, unadjusted analyses showed that in the UK population, the odds of reporting self‐harm were higher among individuals in the second (OR = 1.5; 95% CI = 1.1–1.9) and third (OR = 1.6; 95% CI = 1.2–2.1) lockdowns. However, the highest odds were reported during the lifting of Lockdown 3 (OR = 2.4; 95% CI = 1.9–2.9). Similar effect sizes were found in the adjusted model. Analysis in the second model for both unadjusted and adjusted models demonstrated similar results, with the second and third lockdowns and the lifting of Lockdown 3 being associated with increased odds of reporting self‐harm (Table 4).

**Table 4:** Association of restrictions with self‐harm.

Outcome	Variable	Category	Unadjusted model				Adjusted model		
			OR	*p*	95% CI		OR	*p*	95% CI
Self‐harm	Wave	Lockdown 1 (reference)				Lockdown 1 (reference)			
		Lifting of Lockdown 1	1.0	0.928	0.8–1.3	Lifting of Lockdown 1	1.0	0.962	0.8–1.2
		Lockdown 2	1.5	0.005	1.1–1.9	Lockdown 2	1.5	0.003	1.2–2
		Lifting of Lockdown 2	1.2	0.208	0.9–1.6	Lifting of Lockdown 2	1.2	0.156	0.9–1.7
		Lockdown 3	1.6	0.001	1.2–2.1	Lockdown 3	1.6	0.001	1.2–2.1
		Lifting of Lockdown 3	2.4	0.001	1.9–2.9	Lifting of Lockdown 3	2.4	0.001	1.9–3
	Sex	Male	0.9	0.52	0.9–1.1	Male	1.0	0.548	0.9–1.2
	Age (years)	18–24	15	0.001	12.2–18.5	18–24	11.9	0.001	8.9–15.9
		25–34	8.4	0.001	6.8–10.3	25–34	6.5	0.001	5–8.6
		35–44	5.8	0.001	4.6–7.2	35–44	4.4	0.001	3.3–5.8
		45–54	2.9	0.001	2.3–3.7	45–54	2.1	0.001	1.6–2.9
	Working status	Working part‐time	1.2	0.029	1–1.4	Working part‐time	1.3	0.001	1.1–1.6
		Full‐time student	2.5	0.001	2.1–3.1	Full‐time student	1.1	0.526	0.9–1.4
		Retired	0.2	0.001	0.1–0.2	Retired	0.6	0.028	0.4–1
		Unemployed	2.2	0.001	1.8–2.7	Unemployed	1.8	0.001	1.4–2.2
	Social grade	ABC 1, middle class	0.8	0.001	0.7–0.9	ABC 1, middle class	0.9	0.064	0.8–1
Self‐harm									
	Wave	Lifting of Lockdown 1				Lifting of Lockdown 1			
		Lockdown 2	1.5	0.005	1.1–1.9	Lockdown 2	1.6	0.001	1.2–2.1
		Lifting of Lockdown 2	1.2	0.208	0.9–1.6	Lifting of Lockdown 2	1.2	0.175	0.9–1.6
		Lockdown 3	1.6	0.001	1.2–2.1	Lockdown 3	1.6	0.001	1.3–2.1
		Lifting of Lockdown 3	2.4	0.001	1.9–2.9	Lifting of Lockdown 3	2.1	0.001	1.8–2.5
	Sex	Male	0.9	0.52	0.9–1.1	Male	1.1	0.072	1–1.3
	Age (years)	18–24	15	0.001	12.2–18.5	18–24	11.5	0.001	8.2–16
		25–34	8.4	0.001	6.8–10.3	25–34	6.2	0.001	4.6–8.4
		35–44	5.8	0.001	4.6–7.2	35–44	4.0	0.001	3.0–5.4
		45–54	2.9	0.001	2.3–3.7	45–54	1.8	0.001	1.3–2.4
	Working status	Working part‐time	1.2	0.029	1–1.4	Working part‐time	1.1	0.396	0.9–1.3
		Full‐time student	2.5	0.001	2.1–3.1	Full‐time student	1.0	0.871	0.7–1.3
		Retired	0.2	0.001	0.1–0.2	Retired	0.4	0.001	0.3–0.7
		Unemployed	2.2	0.001	1.8–2.7	Unemployed	0.9	0.577	0.7–1.2
	Social grade	ABC 1, middle class	0.8	0.001	0.7–0.9	ABC 1, middle class	1.0	0.587	0.9–1.2
	Marital status	In a civil partnership	4.5	0.001	3.1–6.4	In a civil partnership	1.6	0.029	1–2.4
		Separated but still legally married or in a civil partnership	3.4	0.001	2.6–4.5	Separated but still legally married or in a civil partnership	1.1	0.718	0.8–1.5
		Living with a partner but neither married nor in a civil partnership	2	0.001	1.6–2.4	Living with a partner but neither married nor in a civil partnership	0.9	0.277	0.7–1.1
		In a relationship but not living together	3.4	0.001	2.7–4.3	In a relationship but not living together	1.0	0.796	0.8–1.4
		Single	2.8	0.001	2.4–3.3	Single	0.9	0.312	0.7–1.1
		Divorced	1.4	0.003	1.1–1.8	Divorced	1.2	0.121	0.9–1.6
		Widowed	0.4	0.004	0.2–0.8	Widowed	1.0	0.895	0.5–1.8
	Pre‐existing mental health condition	Yes	7.3	0.001	6.4–8.3	Yes	3.6	0.001	3–4.3
	Disability/long‐term condition	Yes, limited a lot	7.5	0.001	6.5–8.7	Yes, limited a lot	6.2	0.001	5–7.5
		Yes, limited a little	2.6	0.001	2.2–3	Yes, limited a little	2.2	0.001	1.8–2.7

After analyzing the restriction periods and the related prevalence of self‐harm, it was found that compared to Lockdown 1, the second and third lockdowns were associated with a higher likelihood for people to have self‐harm behaviors; whereas, lifting of lockdowns was also associated with a higher likelihood.

### 3.3 Interaction Effects for Suicidal Thoughts and Self‐Harm

#### 3.3.1 Suicidal Thoughts

##### 3.3.1.1 Interaction for Age

The interaction between Lockdown 3 and the 18–24 (OR = 1.9; 95% CI = 1.2–3.2) and 25–34 (OR = 1.8; 95% CI = 1.2–2.7) age groups was found to be statistically significant. Similarly, the lifting of Lockdown 3 had a significant interaction with the 18–24 (OR = 2.4; 95% CI = 1.7–3.4), 25–34 (OR = 1.8; 95% CI = 1.3–2.5), and 35–44 (OR = 1.4; 95% CI = 1–2) age groups (Table 5). The results indicate that, similar to the pattern of self‐harm, compared to older people, younger adults were especially more likely to have suicidal thoughts at later stages of the pandemic (since Lockdown 3).

##### 3.3.1.2 Interaction for Working Status

Analysis showed that Lockdown 3 had a statistically significant interaction with the full‐time student group (OR = 2; 95% CI = 1.1–3.6), demonstrating that students tended to have more suicidal thoughts during the third lockdown. The interaction between Lockdown 3 and the lifting of Lockdown 3 with the retired group was also significant (OR = 0.4; 95% CI = 0.3–0.6), showing that the retired group was less likely to have suicidal thoughts (Table 6).

**Table 5:** Interaction between lockdown and age (self‐harm).

Self‐harm	Odds ratio	*p* > *z*	95% CI
Wave 2			
Lifting of Lockdown 1	0.9	0.582	0.5–1.5
Lockdown 2	1.2	0.641	0.6–2.4
Lifting of Lockdown 2	1.2	0.693	0.6–2.4
Lockdown 3	0.6	0.216	0.2–1.4
Lifting of Lockdown 3	1.1	0.764	0.6–2
Age group			
18–24	5.9	0.001	3.1–11.2
25–34	4	0.001	2.2–7.6
35–44	3.7	0.001	2.0–6.9
45–54	1.2	0.622	0.6–2.7
Work status			
Working part‐time	1.3	0.002	1.1–1.6
Full‐time student	1.1	0.348	0.9–1.4
Retired	0.6	0.027	0.4–1
Unemployed	1.8	0.001	1.4–2.2
Not working/other	2.3	0.001	1.9–2.7
Gender			
Male	1	0.552	0.9–1.2
Social grade			
ABC1, middle class	0.9	0.063	0.8–1
Wave 2 #agegroup			
Lifting of Lockdown 1 #18–24	1.4	0.416	0.7–2.8
Lifting of Lockdown 1 #25–34	1.2	0.687	0.6–2.4
Lifting of Lockdown 1 #35–44	1	0.897	0.5–2
Lifting of Lockdown 1 #45–54	1.5	0.333	0.6–3.7
Lockdown 2 #18–24	1.4	0.49	0.6–3.4
Lockdown 2 #25–34	1.3	0.595	0.5–3.1
Lockdown 2 #35–44	1.1	0.889	0.4–2.6
Lockdown 2 #45–54	2.1	0.175	0.7–5.9
Lifting of Lockdown 3 #18–24	1.3	0.591	0.5–3.4
Lifting of Lockdown 3 #25–34	1	0.949	0.4–2.6
Lifting of Lockdown 3 #35–44	0.7	0.475	0.3–1.8
Lifting of Lockdown 3 #45–54	1.7	0.324	0.6–5
Lockdown 3 #18–24	4.3	0.007	1.5–12.5
Lockdown 3 #25–34	2.7	0.059	1–7.6
Lockdown 3 #35–44	2.2	0.141	0.8–6.3
Lockdown 3 #45–54	3.5	0.043	1–11.5
Lifting of Lockdown 3 #18–24	3.4	0.001	1.6–7
Lifting of Lockdown 3 #25–34	2.6	0.011	1.2–5.3
Lifting of Lockdown 3 #35–44	1.6	0.232	0.7–3.3
Lifting of Lockdown 3 #45–54	1.9	0.146	0.8–4.8

**Table 6:** Interaction for working status (self‐harm).

Self‐harm	Odds ratio	*p* > *z*	95% CI
Wave 2			
Lifting of Lockdown 1	0.9	0.63	0.6–1.3
Lockdown 2	1.3	0.348	0.8–2
Lifting of Lockdown 3	1.2	0.502	0.7–1.9
6	1.5	0.111	0.9–2.3
7	3.1	0.001	2.2–4.4
Age group			
18–24	11.6	0.001	8.7–15.4
25–34	6.5	0.001	4.9–8.5
35–44	4.4	0.001	3.3–5.8
45–54	2.1	0.001	1.6–2.9
Work status			
Working part‐time	1.1	0.677	0.6–2.1
Full‐time student	1.3	0.465	0.7–2.3
Retired	1.2	0.675	0.5–2.5
Unemployed	2.1	0.044	1–4.4
Not working/other	2.3	0.003	1.3–3.9
Gender			
Male	1	0.8	0.9–1.2
Social grade			
ABC1, middle class	0.9	0.034	0.8–1
Wave 2 #workstatus			
Lifting of Lockdown 1 #Working part‐time	1.4	0.333	0.7–2.9
Lifting of Lockdown 1 #Full‐time student	1	0.939	0.5–2.1
Lifting of Lockdown 1 #Retired	1.1	0.895	0.4–2.5
Lifting of Lockdown 1 #Unemployed	1.1	0.769	0.5–2.6
Lifting of Lockdown 1 #Not working/other	1.1	0.77	0.6–2.1
Lockdown 2 #Working part‐time	1.3	0.537	0.6–3.1
Lockdown 2 #Full‐time student	1.4	0.469	0.6–3.4
Lockdown 2 #Retired	0.8	0.7	0.3–2.5
Lockdown 2 #Unemployed	1.3	0.615	0.5–3.5
Lockdown 2 #Not working/other	1.7	0.184	0.8–3.7
Lifting of Lockdown 3 #Working part‐time	1.5	0.407	0.6–3.7
Lifting of Lockdown 3 #Full‐time student	0.9	0.753	0.3–2.2
Lifting of Lockdown 3 #Retired	0.1	0.079	0.1–1.2
Lifting of Lockdown 3 #Unemployed	1	0.97	0.4–2.7
Lifting of Lockdown 3 #Not working/other	1.5	0.345	0.7–3.4
Lockdown 3 #Working part‐time	1.5	0.349	0.6–3.5
Lockdown 3 #Full‐time student	0.6	0.411	0.2–1.9
Lockdown 3 #Retired	0.6	0.392	0.2–2
Lockdown 3 #Unemployed	1.5	0.395	0.6–4.1
Lockdown 3 #Not working/other	1.1	0.755	0.5–2.4
Lifting of Lockdown 3 #Working part‐time	1.0	0.886	0.5–1.8
Lifting of Lockdown 3 #Full‐time student	0.8	0.434	0.4–1.5
Lifting of Lockdown 3 #Retired	0.2	0.003	0.1–0.6
Lifting of Lockdown 3 #Unemployed	0.5	0.064	0.2–1
Lifting of Lockdown 3 #Not working/other	0.7	0.227	0.4

##### 3.3.1.3 Interaction for Gender

The interaction between the lifting of Lockdown 3 and the male group (OR = 1.5; 95% CI = 1.2–1.9; *p* < 0.001) was found to be statistically significant (Table 7). This suggests that, similar to the pattern of self‐harm, the male group was more likely to have suicidal thoughts in the later stages of the pandemic, compared to the female group.

**Table 7:** Interaction for gender (self‐harm).

Self‐harm	Odds ratio	*p* > *z*	95% CI
Wave 2			
Lifting of Lockdown 1	0.9	0.413	0.7–1.2
Lockdown 2	1.6	0.003	1.2–2.2
Lifting of Lockdown 3	1.1	0.711	0.7–1.5
Lockdown 3	1.2	0.343	0.8–1.7
Lifting of Lockdown 3	1.7	0.001	1.3–2.3
Age group			
18–24	12	0.001	9–16
25–34	6.5	0.001	5–8.6
35–44	4.4	0.001	3.3–5.8
45–54	2.1	0.001	1.6–2.9
Work status			
Working part‐time	1.3	0.001	1.1
Full‐time student	1.1	0.49	0.9
Retired	0.6	0.032	0.4
Unemployed	1.8	0.001	1.4
Not working/other	2.3	0.001	1.9–2.7
Gender			
Male	0.7	0.077	0.5–1
Social grade			
ABC1, middle class	0.9	0.045	0.8–1
Wave 2 #gender			
Lifting of Lockdown 1 #Male	1.3	0.297	0.8–2.1
Lockdown 2 #Male	0.8	0.574	0.5–1.5
Lifting of Lockdown 3 #Male	1.4	0.271	0.8–2.6
Lockdown 3 #Male	1.9	0.03	1.1–3.5
Lifting of Lockdown 3 #Male	2	0.002	1.3–3.2

#### 3.3.2 Self‐Harm

##### 3.3.2.1 Interaction for Age

The interaction between Lockdown 3 and the 18–24 age group was found to be statistically significant (OR = 4.3; 95% CI = 1.5–12.5). Similarly, the interaction between Lockdown 3 and the 45–54 age group was statistically significant (OR = 3.5; 95% CI = 1–11.5). We also found a statistically significant interaction between the lifting of Lockdown 3 and the 18–24 age group (OR = 3.4; 95% CI = 1.6–7) and with the 25–34 age group (OR = 2.6; 95% CI = 1.2–5.3). The results indicate that compared to older people, younger adults were especially more likely to take self‐harm actions at later stages of the pandemic (since Lockdown 3), and the middle‐aged group tended to report more self‐harm during the third lockdown (Table 8).

##### 3.3.2.2 Interaction for Working Status

Analysis revealed a statistically significant interaction between the lifting of Lockdown 3 and the retired working status group (OR = 0.2; 95% CI = 0.1–0.6) (Table 9). The result demonstrates that the retired group was significantly less likely to take self‐harm actions, while other groups were reporting an increase in self‐harm when facing the lifting of Lockdown 3.

**Table 8:** Interaction for age (suicidal thoughts).

Suicidal thoughts	Odds ratio	*p* > *z*	95% CI
Wave 2			
Lifting of Lockdown 1	1	0.771	0.8–1.3
Lockdown 2	1.5	0.004	1.1–1.9
Lifting of Lockdown 3	1.2	0.231	0.9–1.6
Lockdown 3	1.1	0.469	0.8–1.5
Lifting of Lockdown 3	1	0.93	0.8–1.3
Age group			
18–24	3	0.01	2.2–4.2
25–34	2.5	0.01	1.9–3.3
35–44	2.2	0.001	1.6–2.9
45–54	1.5	0.008	1.1–2
Work status			
Working part‐time	1.2	0.001	1.1–1.3
Full‐time student	1.3	0.001	1.1–1.6
Retired	0.8	0.006	0.7–0.9
Unemployed	2.2	0.001	1.9–2.4
Not working/other	2	0.001	1.8–2.2
Gender			
Male	1.1	0.002	1–1.2
Social grade			
ABC1, middle class	0.9	0.001	0.8–0.9
Wave 2 #agegroup			
Lifting of Lockdown 1 #18–24	1.4	0.052	1–2
Lifting of Lockdown 1 #25–34	1.2	0.176	0.9–1.7
Lifting of Lockdown 1 #35–44	1.1	0.535	0.8–1.5
Lifting of Lockdown 1 #45–54	1.1	0.743	0.8–1.5
Lockdown 2 #18–24	1.2	0.449	0.7–1.9
Lockdown 2 #25–34	1	0.882	0.6–1.4
Lockdown 2 #35–44	1	0.81	0.7–1.6
Lockdown 2 #45–54	1.1	0.583	0.7–1.7
Lifting of Lockdown 3 #18–24	1.4	0.198	0.8–2.2
Lifting of Lockdown 3 #25–34	1.4	0.105	0.9–2.1
Lifting of Lockdown 3 #35–44	1.3	0.271	0.8–1.9
Lifting of Lockdown 3 #45–54	1.3	0.176	0.9–2.1
Lockdown 3 #18–24	1.9	0.008	1.2–3.2
Lockdown 3 #25–34	1.8	0.004	1.2–2.7
Lockdown 3 #35–44	1.4	0.103	0.9–2.1
Lockdown 3 #45–54	1.2	0.348	0.8 1.9
Lifting of Lockdown 3 #18–24	2.4	0.001	1.7–3.4
Lifting of Lockdown 3 #25–34	1.8	0.001	1.3–2.5
Lifting of Lockdown 3 #35–44	1.4	0.039	1–2
Lifting of Lockdown 3 #45–54	1.2	0.391	0.8–1.7

**Table 9:** Interaction for working status (suicidal thoughts).

Suicidal thoughts	Odds ratio	*p* > *z*	95% CI
Wave 2			
Lifting of Lockdown 1	1.1	0.129	1–1.4
Lockdown2	1.5	0.001	1.2–1.9
Lifting of Lockdown 2	1.4	0.001	1.1–1.8
Lockdown 3	1.6	0.001	1.3–2
Lifting of Lockdown 3	1.8	0.001	1.5–2.1
Age group			
18–24	4.8	0.001	4.2–5.5
25–34	3.4	0.001	3–3.8
35–44	2.6	0.001	2.3–2.9
45–54	1.7	0.001	1.5–1.9
Work status			
Working part‐time	1.1	0.548	0.8–1.5
Full‐time student	1	0.81	0.7–1.5
Retired	1.2	0.212	0.9–1.6
Unemployed	2.2	0.001	1.5–3.2
Not working/other	2	0.001	1.5–2.6
Gender			
Male	1.1	0.006	1–1.2
Social grade			
ABC1, middle class	0.9	0.001	0.8–0.9
Wave 2 #workstatus			
Lifting of Lockdown 1 #Working part‐time	1.2	0.345	0.8–1.7
Lifting of Lockdown 1 #Full‐time student	1.4	0.138	0.9–2.1
Lifting of Lockdown 1 #Retired	0.8	0.228	0.6–1.1
Lifting of Lockdown 1 #Unemployed	1.1	0.793	0.7–1.6
Lifting of Lockdown 1 #Not working/other	1.1	0.716	0.8–1.5
Lockdown 2 #Working part‐time	1	0.948	0.7–1.5
Lockdown 2 #Full‐time student	1.2	0.606	0.7–2.1
Lockdown 2 #Retired	0.8	0.238	0.5–1.2
Lockdown 2 #Unemployed	1.1	0.62	0.7–2
Lockdown 2 #Not working/other	1.3	0.234	0.8–2
Lifting of Lockdown 3 #Working part‐time	1.1	0.591	0.7–1.8
Lifting of Lockdown 3 #Full‐time student	1.2	0.536	0.7–2.1
Lifting of Lockdown 3 #Retired	0.7	0.161	0.5–1.1
Lifting of Lockdown 3 #Unemployed	1.2	0.552	0.7–2
Lifting of Lockdown 3 #Not working/other	1.3	0.201	0.9–2
Lockdown 3 #Working part‐time	0.9	0.556	0.6–1.4
Lockdown 3 #Full‐time student	2	0.019	1.1–3.6
Lockdown 3 #Retired	0.6	0.042	0.4–1
Lockdown 3 #Unemployed	1.2	0.539	0.7–2
Lockdown 3 #Not working/other	0.9	0.56	0.6–1.3
Lifting of Lockdown 3 #Working part‐time	1	0.862	0.7–1.4
Lifting of Lockdown 3 #Full‐time student	1	0.857	0.7–1.6
Lifting of Lockdown 3 #Retired	0.4	0.001	0.3–0.6
Lifting of Lockdown 3 #Unemployed	0.7	0.128	0.5–1.1
Lifting of Lockdown 3 #Not working/other	0.8	0.105	0.5–1.1

##### 3.3.2.3 Interaction for Gender

Analysis showed a statistically significant interaction between Lockdown 3 and the male group (OR = 1.9; 95% CI = 1.1–3.5) and also the lifting of Lockdown 3 with the male group (OR = 2; 95% CI = 1.3–3.2) (Table 10). This result suggests that the male group was more likely to have self‐harm behaviors in the later stages of the pandemic, compared to the female group.

**Table 10:** Interaction for gender (suicidal thoughts).

Suicidal thoughts	Odds ratio	*p* > *z*	95% CI
Wave 2			
Lifting of Lockdown 1	1.2	0.012	1–1.4
Lockdown 2	1.5	0.001	1.3–1.8
Lifting of Lockdown 3	1.5	0.001	1.3–1.8
Lockdown 3	1.4	0.001	1.2–1.7
Lifting of Lockdown 3	1.2	0.005	1.1–1.4
Age group			
18–24	4.9	0.001	4.3–5.6
25–34	3.4	0.001	3.1–3.9
35–44	2.6	0.001	2.3–3
45–54	1.7	0.001	1.5–1.9
Work status			
Working part‐time	1.2	0.001	1.1–1.3
Full‐time student	1.3	0.001	1.1–1.5
Retired	0.8	0.009	0.7–1
Unemployed	2.2	0.001	1.9–2.4
Not working/other	2	0.001	1.8–2.2
Gender			
Male	1	0.708	0.8–1.2
Social grade			
ABC1, middle class	0.9	0.001	0.8–0.9
Wave 2 #gender			
Lifting of Lockdown 1 #Male	1	0.992	0.8–1.2
Lockdown 2 #Male	1.1	0.609	0.8–1.4
Lifting of Lockdown 3 #Male	1	0.952	0.8–1.3
Lockdown 3 #Male	1.2	0.127	0.9–1.7
Lifting of Lockdown 3 #Male	1.5	0.001	1.2–1.9

## 4 Discussion

The main objective of the study was to investigate how various COVID‐19‐related restrictions affected suicidal thoughts and self‐harm in UK adults throughout the pandemic, with the aim of aiding the design of appropriate targeted public mental health interventions and preventative implementation measures.

The study has found that, during the COVID‐19 pandemic and related public health restrictions, there has been a general upward trajectory over time of suicidal thoughts (Table 1) and self‐harm (Table 3) amongst specific groups, even during the lifting of lockdowns. Particularly at later stages of the pandemic, with repeated lockdowns and lifting of lockdowns, where people might have become tired of a seemingly endless cycle and feel stressed and more unbearable about the future to come (Tables 2 and 4). Corresponding to the main objective and the specific sub‐objectives of the study, Figures 2, 3, 4, 5, 6, 7, 8, 9, 10, 11, 12, 13, 14, 15 have not only shown the overall trends of prevalence of suicidal thought and self‐harm among the overall population, as well as the differences among diverse population groups across the various pandemic phases included in the study. Most evidently, younger people (aged 18–24) were experiencing higher prevalence and odds of reporting suicidal thoughts and self‐harm. These increases were also especially concentrated in people reporting a pre‐existing mental health condition and disabilities. As shown from the multivariate analysis results in Tables 1, 2, 3, 4, the changing trajectories of suicidal thoughts and self‐harm were generally upward, even in the event of lifting lockdowns. When taking a closer look at the specific groups, the results indicate that younger adults were especially more likely to have suicidal thoughts (Table 5) and self‐harm behaviors (Table 8) at later stages of the pandemic (since Lockdown 3), while the middle‐aged group tended to report more self‐harm during the third lockdown (Table 8). The retired group was significantly less likely to have suicidal thoughts (Table 6) and take self‐harm actions (Table 9), while other employment groups were reporting an increase in self‐harm when facing the lifting of Lockdown 3. The male group was more likely to have suicidal thoughts and self‐harm in the later stages of the pandemic, compared to the female group.

It is noteworthy that some studies on the effect of other epidemics on suicide mortality found an increase (at least in some age groups) in suicidality during epidemics [32, 33, 34]. By contrast, other investigations have given rise to doubt concerning the elevation of suicidality during epidemics/pandemics. Sometimes the results are contradictory, even regarding the effects of the same pandemic [9, 10, 32, 35].

Furthermore, the researchers found that individuals with pre‐existing mental health conditions or disabilities were at higher risk of experiencing suicidal thoughts and self‐harm because of the COVID‐19 pandemic. A possible explanation for this is that the pandemic has led to increased isolation and reduced access to support services, making it harder for people who suffer from pre‐existing mental ill‐health or other long‐term conditions to deal with hardship and uncertainty [36]. A study in Canada following a similar methodology found that those with a pre‐existing mental ill‐health and those identifying as LGBTQ+ were more likely to report suicidal thoughts/feelings and self‐harm during the country's first lockdown, as were indigenous peoples, people with a disability, and those with low household income [37]. Finally, the results from the study also support longitudinal analysis from 15 countries in which more stringent mobility restrictions were later associated with adverse mental health outcomes in the population [38]. However, the pandemic's impacts on suicidal thoughts, self‐harm, or suicidal behaviors may be much longer‐lasting, warranting follow‐up studies to explore the long‐term mental health impacts of the pandemic.

## 5 Conclusion

Findings of increased prevalence of suicidal thoughts and self‐harm amongst young people are consistent with evidence from Owens et al. [39], which suggests that lockdown caused a well‐being crisis in young people. Shi et al. [40] reported that young adults aged 18‐24 had almost twice the odds of having suicidal thoughts than older groups (41–50 and 51–60 years of age). The results also indicated that the 55+ age group had the lowest odds of suicidal thinking and self‐harming behavior.

This study highlights the significant mental health challenges posed by the COVID‐19 pandemic, with a notable upward trajectory in suicidal thoughts and self‐harm across various population groups, even following the lifting of lockdowns. The findings indicate that the mental health burden of the pandemic did not subside with the easing of restrictions, and in some cases, worsened over time, especially in response to repeated lockdowns and uncertain future conditions.

In contrast to other studies that suggest mixed results regarding the impact of pandemics on suicidality [9, 35], our data point to a clear increase in suicidality and self‐harm during the pandemic's later stages. Interestingly, retired individuals showed lower rates of suicidal thoughts and self‐harm, suggesting that retirement might have buffered some of the psychological impacts of lockdowns compared to more active or employed groups. Conversely, the male population appeared to face greater mental health challenges during the pandemic's later stages, indicating the need for gender‐sensitive interventions.

Although the restrictions in the United Kingdom were lifted in July 2021, it remains a near‐certainty that another pandemic and associated restrictive measures might occur at some point in the future. The evidence can inform not only future action in the United Kingdom, in terms of pandemic mental health preparedness, but also the approaches of other countries around the world. In this context, it is noteworthy that measures to protect mental health do not need to be in conflict with measures to curb the spread of a pandemic. Rather, using this evidence, the UK and other governments should shape holistic policy and community public health responses to pandemics, and be ready to quickly mobilize extra resources to mitigate the negative mental health effects of pandemic‐related lockdown‐type restrictions.

Overall, these findings underscore the importance of early identification and targeted mental health interventions, particularly for vulnerable groups, during both current and future public health crises. It is crucial to recognize that the mental health impact of the COVID‐19 pandemic may be prolonged, and further longitudinal studies are essential to assess the long‐term consequences of these disruptions on suicidal behaviors, self‐harm, and general well‐being.

### 5.1 Limitations

Although the study sheds light on the pandemic's impact on suicidal thoughts and self‐harm behaviors, the researchers acknowledge that there are limitations. The researchers utilized online recruitment methods of participant sampling to widen access to a large sample size. This was a useful approach during the pandemic and lockdowns, but it has its limitations, such as selection bias, as it narrows the sample to those with internet access. Therefore, key demographics may be underrepresented, such as the elderly, lower‐income individuals, and people living in rural/remote/internet‐deprived areas [41, 42]. Surveys are also open to response bias, whereby individuals may provide inaccurate answers as a function of social‐desirability bias or confirmation bias. Moreover, the researchers did not collect pre‐lockdown baseline data on suicidality and self‐harm specifically, exacerbating the challenge of determining causality. National suicide prevention strategies should include real‐time suicide surveillance, which can significantly improve suicide monitoring, strengthen policy, guide investment planning, and enable more timely responses and actions to address identified risk factors. Further, this research was conceptualized, approved, commenced in February–March 2020, and conducted prior to the new DSM‐5‐TR publication and its subsequent full operationalization. Consequently, the research was carried out against the backdrop of the previous DSM‐TR version. Any future research should keep the new DSM‐5‐TR changes or ICD‐11 in mind.

### 5.2 Policy Implications

The study has shown that suicidal thoughts and self‐harm can be relatively common during an epidemic/pandemic, especially for certain population sub‐groups and depending on contextual factors and measures. Even if increased suicidal thoughts did not lead to an actual increase in deaths by suicide, the experience of suicidality and self‐harm is in itself really important from a public health perspective, as it is a proxy for “living in misery” and closely linked to reduced life satisfaction and emotional suffering [43]. Hence, stakeholders should use this evidence to assess and address the multiple and complex causes of suicidality and low well‐being.

Action should be considered across the social determinants of mental health and factors of social inequality by providing financial support to those in need and addressing the treatment gap resulting from underfunded mental health services to help meet the demand of a possible post‐pandemic mental health crisis [44]. Community interventions that promote connectedness and kindness will also need to be supported, especially during infectious‐disease outbreak periods (or health crises), as they are associated with nurturing hope and human dignity [45].

Further, a key policy priority should be to plan for long‐term suicide prevention strategies using the principles of proportionate universality [46]. The study provides evidence on what the highest risk groups can be for targeted interventions. Preventive measures can increase awareness about suicide and mental health, which can help to promote help‐seeking behavior and remove barriers to support and care. The pandemic affected people's lives and lifestyles differently across the world; therefore, a tailored approach to supportive interventions and advice is required across populations to provide appropriate strategies for aiding people to rebuild a balanced lifestyle pattern [47].

The coronavirus pandemic might not have had an immediate effect on suicide rates and attempts; however, taking into consideration the exacerbated risk factors related to the pandemic, there is a possibility that suicide attempts and rates could increase in the long‐term [48, 49]. Historically, similar lag effects of exposure to stressful situations on suicide rates have been observed after World War I and II and Spanish Influenza, and after the global economic crisis of 2008 [50]. A critical finding of the study was that rates of suicidal thoughts and self‐harm were higher at subsequent stages of lockdowns (Lockdowns 2 and 3, rather than Lockdown 1), which may be attributed to stressors such as loneliness, extended job insecurity, bereavement, physical illness, and a compounding effect over time. They were also higher after the lifting of Lockdown 3, further indicating a cumulative impact effect over the time and on‐and‐off restrictions. This suggests that sustained early action to curb the spread of the coronavirus, and clear communication and clarity around measures, would have had a more positive effect on mental health, rather than the well‐being measures that followed the multiple (and various forms of) lockdowns. This is consistent with epidemiological evidence, which suggests that countries that took decisive action early were able to reduce the burden of severe disease and premature death [51].

At the same time, it remains important to provide support for people living in suicidal distress. It is noteworthy that the health and socio‐economic consequences will last beyond the pandemic [3]. For example, it has been proposed that taking a strategy of addressing suicidal behavior as an independent construct could harmonize clinical and research data and permit the leveraging of large health‐related databases to discover clinical and social markers of imminent and longer‐term suicide risk. Therefore, as part of healthy communities, societies, and also pandemic preparedness, it is important that relevant stakeholders create appropriate strategies to help affected populations and promote togetherness and community cohesion as key protective factors for people's mental health.

## Author Contributions

C.L., with help from L.G., wrote the initial draft manuscript. T.V.B., L.G., S.M. revised the initial draft and contributed towards all subsequent iterations of the manuscript. T.V.B. and A.A.K. are joint study leads. A.M. and G.D. are lead collaborators. S.S. is the study coordinator. C.L., D.C.‐K., S.M., L.G., M.B., and L.T. are researchers on the study. C.L. and TVB led the qualitative work. L.G. led the quantitative work. C.L., L.G., T.V.B., S.M. carried out all the manuscript revisions. All authors reviewed the manuscript for important intellectual content and approved the final version of the manuscript.

## Conflicts of Interest

The authors declare no conflicts of interest.

## Transparency Statement

The lead author Tine Van Bortel affirms that this manuscript is an honest, accurate, and transparent account of the study being reported; that no important aspects of the study have been omitted; and that any discrepancies from the study as planned (and, if relevant, registered) have been explained.

## Data Availability

The researchers intend to make the survey data available upon completion of the overall “Mental Health in the Pandemic” study and dissemination of all the main findings. The platform through which the researchers aim to do this is DATAMIND (Health Data Research UK): https://www.hdruk.ac.uk/helping-with-health-data/health-data-research-hubs/datamind/.
